# Determination of HU override for gold fiducial markers in proton therapy

**DOI:** 10.1002/acm2.70643

**Published:** 2026-05-28

**Authors:** Xinxin Zhang, Matthew Andriotty, Ning J Yue, Ke Nie, Yin Zhang, Rihan Davis, Rahul Parikh, Zhenyu Xiong, Chi Ma, Xiao Wang

**Affiliations:** ^1^ Department of Radiation Oncology Rutgers Cancer Institute of New Jersey Rutgers Robert Wood Johnson Medical School New Brunswick New Jersey USA

**Keywords:** fiducial Markers, HU Override, proton therapy

## Abstract

**Background:**

In radiotherapy, it is not uncommon to treat patients with metallic implants. Those implants often cause significant amount of artifacts on the simulation CT images, and the intrinsic Hounsfield unit (HU) values cannot be directly used as those of the implant without appropriate evaluations. The alternative solution is to delineate the implant and assign certain HU values to the delineated volume to represent the implant. However, the artifacts make it almost impossible to precisely delineate the sizes and shapes of the metallic implants on the CT images, especially for implants with irregular shapes. It is almost always the case that the delineated “implant” contains both implant itself and adjacent tissues. If the inaccurately delineated implant is considered as the pure implant material, dose calculation around the implant is not accurate, leading to distorted dose distribution (from the real) and misguided treatment plan, especially in proton therapy.

**Purpose:**

This study presents a generalizable method for properly calculating the HU override of small metal implants for proton therapy and evaluated the dosimetric effects of this method as applied to gold fiducial markers in a prostate proton treatment plan.

**Methods:**

The HU value for gold fiducial markers was calculated using a mass‐weighting approach to determine the relative stopping power of the delineated fiducial volumes, taking into account the amounts of gold and tissue within the volumes. This stopping power was used to select a corresponding HU number from the CT calibration curve for dose calculation. To evaluate the effects of this HU override on dosimetry calculation for proton therapy, dose distributions were calculated for a prostate plan with gold fiducial markers in four different ways: with the fiducial contours overridden using the method described in this work, with no override using the intrinsic HU values across the CT images, with delineated fiducial structure overridden based on the assumption that the entire contoured volume was gold, and with modeled fiducial structure which matched as the vendor's design inserted onto the CT images. The fourth method was considered as the closest to reality and as the reference. Further investigations were also conducted to determine the validity of the method and its robustness to inter‐user contouring variation.

**Results:**

Compared to the reference, the non‐overridden and overridden delineated fiducial volumes with pure gold material resulted in inaccurate distortions of the isodose lines in and around the target and considerable unreal reductions in target coverage, while the tissue weighted approach presented in this study yielded the closest resemblance to the reference. This method was also shown to be robust to delineation variations caused by different users.

**Conclusion:**

A method is proposed and validated for tackling the dose calculation challenges imposed with the presence of metallic implants inside patients during radiotherapy planning process. This developed method has the potential to be generalized for other types of metallic materials.

## 1 INTRODUCTION

Fiducial markers are commonly used in image‐guided prostate radiotherapy for their imaged visual clarity to improve setup reproducibility and reduce localization errors compared to soft tissue alignment alone. Multiple studies have demonstrated that fiducial‐based localization reduces inter‐ and intra‐fractional setup variability, thereby improving the accuracy of treatment delivery.^1^, ^2^, ^3^ These markers typically measure 1.0–1.6 mm in diameter and 3–5 mm in length, comparable in size to a grain of rice, and are most often made of gold (density of about 19.32 g/cm^3^). Depending on manufacturers, those fiducials may be produced as tiny rods, spheres or cylinders. Because of their small dimensions, variable orientation once placed inside patient, and high density, accurate delineation of their volume and geometry on computed tomography (CT) is often difficult. In addition, metal‐related CT artifacts such as streaking, beam hardening, and photon starvation lead to distorted Hounsfield Unit (HU) values in the vicinity of the marker, degrading both delineation accuracy and dose calculation reliability,^4^, ^5^, ^6^, ^7^, ^8^ particularly in the case of proton therapy^9^, ^10^. In clinical practice, careful adjustment of CT window and level settings is often needed to optimize delineation accuracy in the presence of metallic implants.

In proton therapy, the relative proton stopping power (RSP) of tissues and implanted materials with respect to water is used in dose calculation. The treatment planning system (TPS) relies on a calibration curve relating to CT HU values to RSP. For high‐*Z* materials such as gold, besides the artifact related issues, their HU values often exceed the calibration limits, leading to erroneous RSP assignments. Such errors can lead to incorrect prediction of proton range, potentially resulting in target underdosage or unintended irradiation of adjacent normal tissue.^11^ Double scattering proton delivery further amplifies these risks as sharp gradients in compensators can impact treatment accuracy due to set up uncertainties.

Previous studies have explored approaches to address these uncertainties introduced by the presence of the metallic materials, including the use of dual‐energy CT to improve RSP prediction^12^, ^13^ and the implementation of Monte Carlo simulations for metallic implants.^10^ Vilches‐Freixas et al. investigated practical considerations for handling different types of implants in proton therapy with a Mevion S250i Hyperscan proton system, including manual HU overrides applied in clinical TPS.^14^ However, the evaluation of fiducial‐specific HU overrides, particularly for small gold markers in prostate cancer treatment, remains limited. The present study addresses this gap by (1) deriving an appropriate RSP for gold fiducial markers; (2) evaluating the dosimetric effects of our proposed HU override in comparison with non‐override (default intrinsic HU) and improperly overridden (simple overridden HU) scenarios in clinical proton treatment plans; and (3) assessing the robustness of the proposed RSP calculation method.

## 2 METHODOLOGY

### 2.1 Energy selection

**FIGURE 1:**
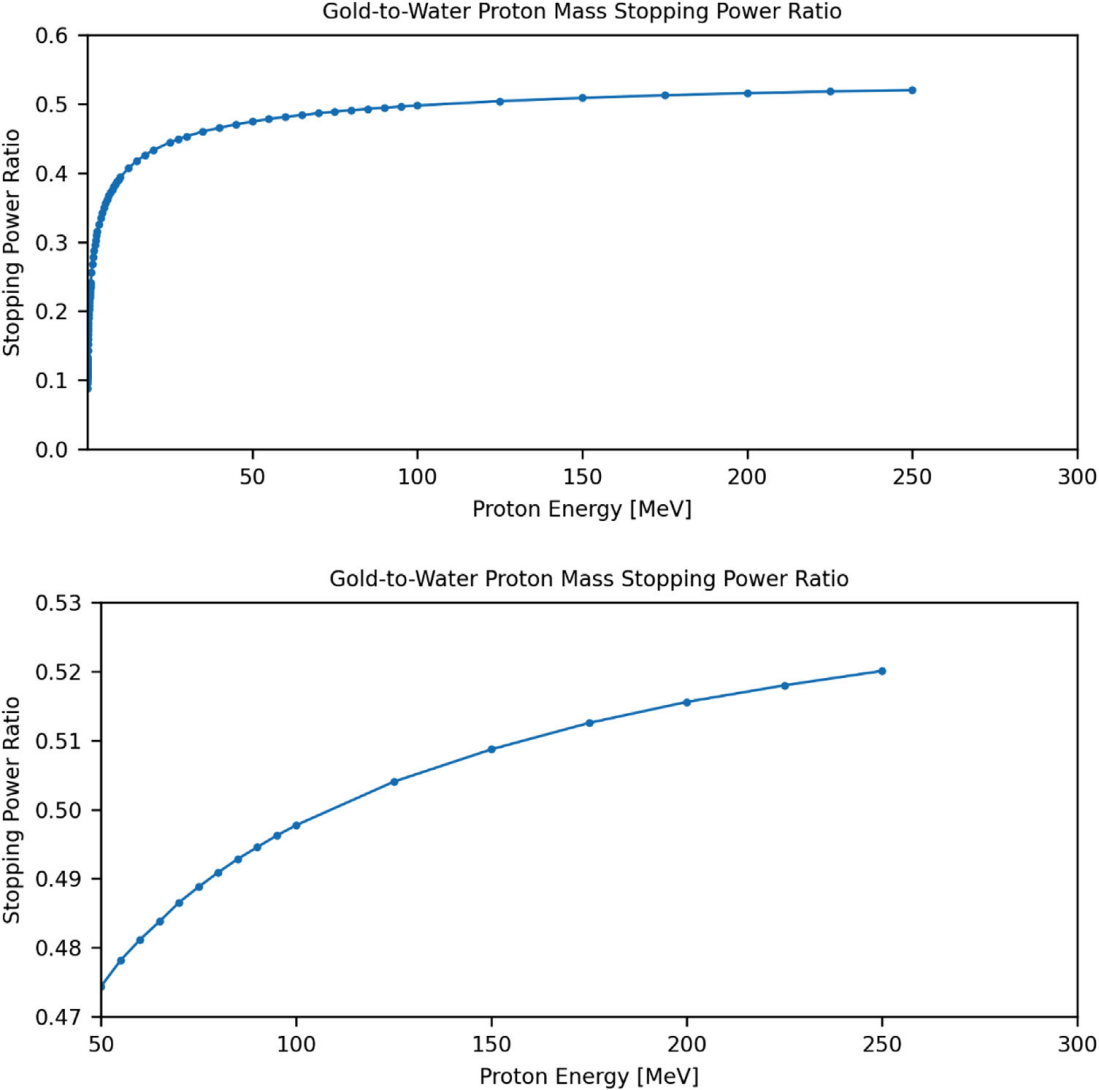
Gold‐to‐water mass stopping power ratios as a function of proton energy 0–250 MeV (top) and 50–250 MeV (bottom).

The Mevion (Mevion Medical Systems, Inc., Littleton, MA) S250 double‐scattering proton machine generates proton beams of up to 250 MeV, corresponding to a maximum penetration depth of approximately 38 cm in water equivalent tissue. In clinical operation, usable proton energies at isocenter typically range from 70 to 250 MeV, yielding penetration depths of approximately 5 to 32 cm in water equivalent tissue after modulation in the beamline. However, the required therapeutic energies depend strongly on target depth. For prostate treatment, where the target lies at a water equivalent depth of approximately 10–15 cm, proton energies from 150 to 230 MeV are typically employed, depending on patient anatomy and beam arrangement.^15^ Spread‐out Bragg peaks (SOBP) are generated by modulating the incident energy distribution to conform to the treatment target volume and depth. To select an appropriate energy for stopping power calculations, the mass proton stopping power of gold and water were obtained from PSTAR (Physical and Nuclear Stopping Power and Range Tables for Protons) database (Physical Measurements Laboratory, National Institute of Standards and Technology)^16^ over the energy range of 1 keV to 250 MeV. The relative mass stopping power of gold with respect to water (RMSP) is calculated as:

(1) $$RMSP={\left(S/\rho \right)}_{gold}\left(E\right)/{\left(S/\rho \right)}_{water}\left(E\right)\;$$

where ${\left(S/\rho \right)}_{gold}$ and ${\left(S/\rho \right)}_{water}$ represent the proton mass stopping power of gold and water at energy E, respectively. As shown in Figure 1, the RMSP exhibits a steep increase at low energies from 1 keV to 20 MeV, followed by a gradual decline between 20–125 MeV, and approaches a plateau beyond 125 MeV. Within the clinically relevant therapeutic energy interval of 150 to 250 MeV, the variation in RMSP was within 2%. Therefore, the average energy of 200 MeV was selected as a representative energy for subsequent stopping‐power calculations and HU override implementation. To validate this energy selection, a broader energy range, 100 to 250 MeV was also evaluated for RMSP, yielding a variation of approximately 4% with an average energy of 175 MeV. This energy was also used for RSP and dose calculation as a comparison.

### 2.2 Determination of proton stopping power in a clinical prostate proton treatment case

The proton treatment plan of a patient diagnosed with stage IIB prostate cancer, treated with moderate hypofractionation to 70 Gy in 28 fractions to the prostate and seminal vesicle, was retrospectively analyzed. Treatments were delivered on a Mevion S250 double‐scattering proton therapy system. Prior to simulation, three cylindrical gold fiducial markers (manufacturer: Civco Medical Solutions, Coralville, IA) with density of 19.32 g/cm^3^, diameter of 1.2 mm, length of 3 mm and volume of 0.0034 cm^3^ were implanted into the prostate to facilitate image‐guided localization. Simulation imaging was performed using a Philips Big Bore RT (Philips Medical System, Netherlands) CT scanner. Axial scans were acquired at 120 kVp with 1.0 mm slice thickness. This scanner provides a 16‐bit image depth, allowing HU values to be represented over a dynamic range of up to 12 000. A site‐specific HU‐to‐RSP calibration curve was commissioned for proton dose calculation using the stoichiometric method of Schneider et al.,^17^ which models elemental composition of calibration material to establish HU‐to‐RSP relationships. The orthopedic metal artifact reduction (O‐MAR) algorithm was applied to reduce image artifacts induced by the fiducials, yielding improved image quality.

Fiducial markers were manually delineated using the Eclipse TPS (Varian Medical Systems, a Siemens Health Company, Palo Alto, CA). A window level range of approximately 187 to 1247 HU was applied to enhance visualization. This setting minimized the appearance of soft tissue and imaging artifacts, allowing the markers to appear with high contrast for delineation. However, due to their small size relative to the CT voxel dimensions and their high‐*Z* composition, partial‐volume averaging and saturation effects prevented faithful representation of the true marker geometry. Consequently, the delineated structure for each of the markers in TPS encompassed both the fiducial marker and small volumes of adjacent tissues, rather than the exact physical location, shape, and size of the marker. Thus, simple assignment of RSP of gold to this structure is not accurate and will lead to misleading dose distributions.

To consider both materials (fiducial material and tissues), which were inadvertently delineated into the structure as the representation of the gold fiducial marker, in the calculation of proton stopping power, the mass ratio of the fiducial material ($f_{fiducial}$) and tissues ($f_{tissue}$) within the delineated structure can be first determined by using the vendor‐provided volume ($V_{fiducial}$) and density ($\rho_{tissue}$) of the fiducial markers and the delineated structure volume ($V_{TPS}$) from the TPS (Equations (2a), (2d)) as

(2a) $$\;m_{tissue}=\left(V_{TPS}-V_{fiducial}\right)\rho_{tissue}$$

(2b) $$\;m_{fiducial}=V_{fiducial}\;\rho_{fiducial}$$

(2c) $$\;f_{tissue}=m_{tissue}/\left(m_{fiducial}+m_{tissue}\right)\;$$

(2d) $$\;f_{fiducial}=m_{fiducial}/\left(m_{fiducial}+m_{tissue}\right)\;$$

The proton mass stopping power of the delineated structure was then calculated as a mass‐weighted average stopping power of a two‐component material^18^: gold and tissue (Equation 3), where the mass stopping power of each component was obtained from PSTAR, as

(3) $$\begin{array}{cc} \; & {\left(S/\rho \right)}_{contoured\;stucture}=f_{fiducial}\;{\left(S/\rho \right)}_{fiducial}\\ & +f_{tissue}{\left(S/\rho \right)}_{tissue} \end{array}$$

This mass stopping power was further used to calculate the stopping power of the delineated structure by multiplying the density of the structure, where the density is the ratio of the total mass (sum of $m_{tissue}$ and $m_{fiducial}$) to the volume (as derived from TPS). Lastly, the relative stopping power (RSP) of the delineated structure with respect to water can be calculated (Equation 4) and used for the selection of a corresponding HU value from the established CT calibration curve to override the intrinsic HU values of the structure for dose calculation. In this study, prostate tissue is assumed to be water for the purposes of stopping power calculation, as

(4) $$\begin{array}{cc} \;RSP & =S_{water}^{contoured\;structure}\\ & =\rho_{structure}{\left(S/\rho \right)}_{structure}/\rho_{water}{\left(S/\rho \right)}_{water}\; \end{array}$$

### 2.3 Dosimetric evaluation in TPS

To evaluate the dosimetric impacts of different approaches of the HU overrides/selections for gold fiducial markers on proton dose distributions, three scenarios were analyzed using the Eclipse TPS with proton convolution superposition (PCS) algorithm. This algorithm is clinically commissioned and currently implemented in our TPS for routine proton therapy planning:

1. Proposed HU—Fiducial marker structures were overridden using the HU values selected to match the tissue weighted RSP as described above.
2. Default intrinsic HU—The fiducials retained their intrinsic CT HU values without any override, and the RSP was assigned directly from the HU‐to‐RSP calibration curve.
3. Simple overridden HU—The entire delineated fiducial structure was assumed to be composed of solid gold. A HU value of 10 000 was assigned to match the physical mass density of gold (19.32 g/cm^3^).

A plan was designed and computed, with the proposed HU for the gold fiducial markers, using bilateral oblique opposed double‐scattered proton beams, with beam parameters including gantry angles, snout position, aperture block shape, and compensator to achieve desired clinical target coverage and organ‐at‐risk sparing. To enable direct dosimetric comparisons, this same plan, preserving all other identical beam and device parameters, was used to re‐compute the dose distribution under scenarios 2 (default intrinsic HU for fiducial markers) and 3 (simple overridden HU for fiducial markers).

### 2.4 Robustness of stopping power calculation

Given that fiducial marker delineation can be affected by inter‐user variability and even the window level setting, it is important to assess the robustness of the proposed method for stopping power calculation under realistic clinical uncertainties.

To establish benchmark dose distributions for the purpose of validating and comparing the distribution differences with the three different fiducial marker overridden approaches, the actual fiducial makers imaged on the CT and related artifacts were first overridden with water equivalent material. Then three structures were modeled and constructed exactly as the manufacturer‐specified fiducial maker design and dimension, in terms of shape, size, and geometry, and were inserted onto the planning CT images at the locations of the three fiducial makers. The orientation and location of each of the model markers were verified via the orthogonal kV images acquired during treatment. A CT value of 10 000 HU was assigned to modeled marker material to match the physical mass density of gold. Voxels immediately adjacent to the markers, which appear artificially hyperdense on CT images due to partial‐volume effects, and the imaging artifacts were contoured and assigned a soft tissue value of 40 HU. The same treatment plan described in Section 2.3 was used to recompute the dose distribution under this scenario. Since this plan represents the scenario in which the fiducial markers were precisely modeled using their true physical dimensions, geometry, and material density, as well as implanted orientation inside patient, the computed dose distributions were near reality and it was therefore designated as the reference plan and used to compare the dose distributions calculated from the plan with HU overrides implemented using the proposed method and other methods in this study.

To further assess robustness with respect to inter‐user variability in fiducial delineation, a 1 mm margin was isotropically added to the original fiducial marker contours, ensuring no overlap with adjacent image artifacts, to simulate the inter‐user delineation variability. This expansion changed the mass ratio between the fiducial and water by including more surrounding tissues (water), which is expected to result in a lower RSP. The adjusted RSP was subsequently used for dose calculations to evaluate the dosimetric impact.

In addition, due to the lack of a well‐defined elemental composition for prostate tissue, water was used as a surrogate material for RSP calculations in this study. To evaluate the degree of the approximation of this assumption, two additional tissue types—striated muscle (density of 1.04 g/cm^3^) and adipose tissue (density of 0.92 g/cm^3^)—were selected to represent the upper and lower bonds of prostate tissue densities, respectively. These tissues were used to substitute for water in the RSP calculations to assess the impact of material selection on the resulting RSP values. To further investigate the range sensitivity to the fiducial RSP and HU override, the beamline properties of the clinical plan were recalculated for different HU values, while maintaining the same gantry angle and aperture shapes. The resulting range differences were compared across the assumptions described above to assess the clinical significance of the HU override strategies.

## 3 RESULTS

### 3.1 Proton stopping power and dosimetric evaluation

Table 1 shows the values of volume, density and mass for the actual fiducial markers, tissues (water) enclosed inside the delineated fiducial structure, and the entire delineated fiducial structure in the TPS for one of the gold fiducial markers in the investigated clinical case. These values were either provided by vendor, derived directly from the TPS or computed as presented in Equations 2a and 2b.

**TABLE 1:** Physical characteristics of gold fiducial markers, enclosed tissue (water) and the contoured structure in the TPS.

	Fiducial	Tissue (water)	Contoured structure
Volume (cm^3^)	0.0034	0.0466	0.0500
Density (g/ cm^3^)	19.32	1.00	2.25
Mass (g)	0.066	0.047	0.112

Table 2a and b lists the mass weight fractions of the gold and water within the delineated fiducial structure (as in Equations 2c and 2d), along with the corresponding mass stopping power, stopping power, and relative stopping power to water for 200 MeV and 175 MeV proton beams (Equations 3 and 4). The computed RSP values for the delineated fiducial markers were 1.608 and 1.604 for 200 MeV and 175 MeV, respectively. Based on the preestablished HU‐to‐RSP CT calibration curve, the corresponding HU values were 1100 and 1092, respectively. As shown, this difference has minimal impact on the dose distribution; thus, 200 MeV is an appropriate energy for stopping power calculation in this study.

**TABLE 2a:** Mass weight and stopping powers of gold fiducials, water and the contoured structure for a proton energy of 200 MeV.

	Mass weight (%)	Mass stopping power (MeV cm^2^/g)	Stopping power (MeV/cm)	Relative stopping power to water
Gold fiducials	58.5%	2.316	44.75	9.961
Tissue (water)	41.5%	4.492	4.492	1.000
Contoured structure	100%	3.220	7.224	1.608

**TABLE 2b:** Mass weight and stopping powers of gold fiducials, water and the contoured structure for a proton energy of 175 MeV.

	Mass weight (%)	Mass stopping power (MeV cm^2^/g)	Stopping power (MeV/cm)	Relative stopping power to water
Gold fiducials	58.5%	2.513	48.55	9.902
Tissue (water)	41.5%	4.903	4.903	1.000
Contoured structure	100%	3.506	7.865	1.604

**FIGURE 2:**
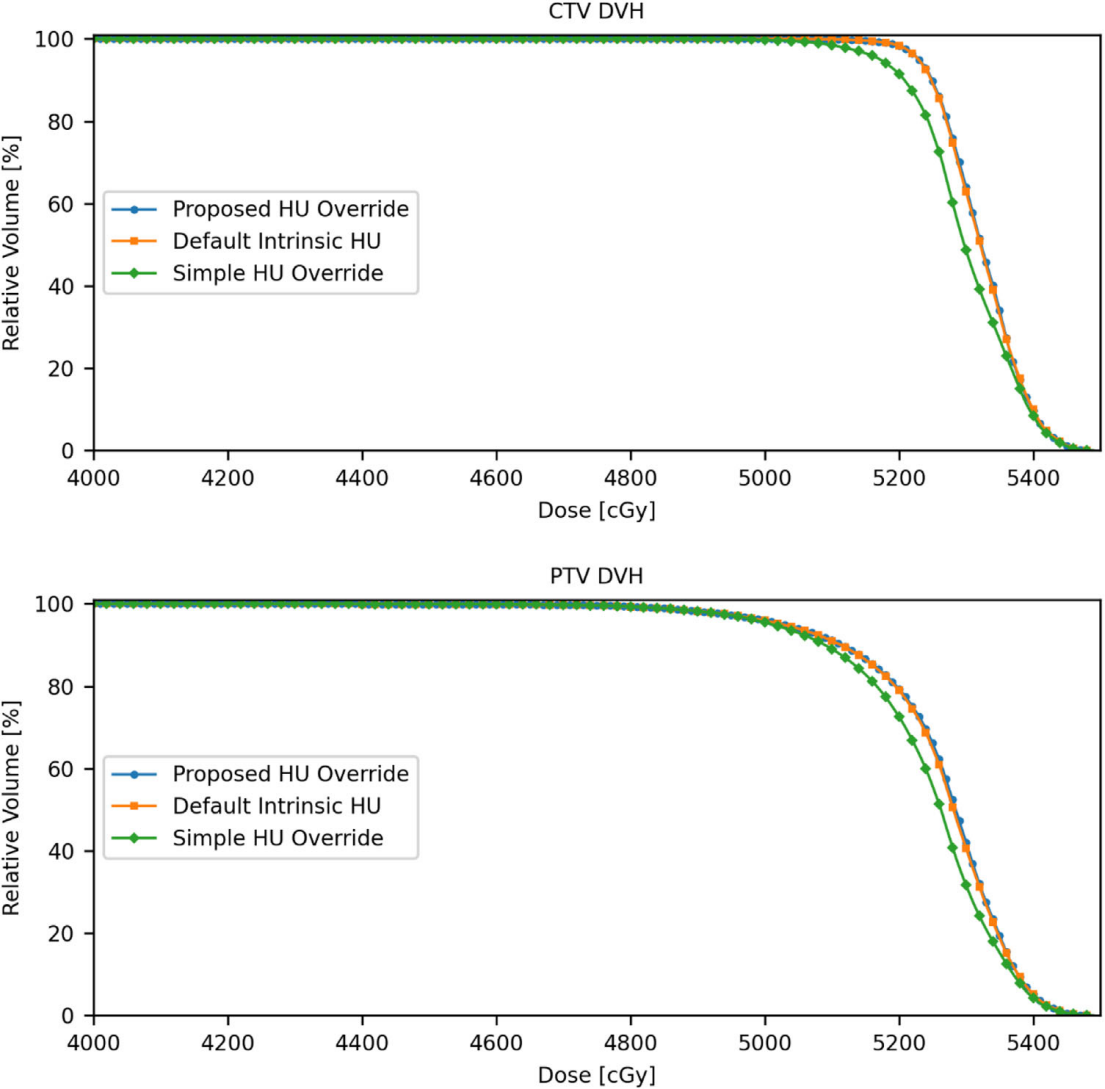
Dose‐volume histograms for the CTV (top) and PTV (bottom) for three different fiducial HU override scenarios.

Figure 2 shows the dose volume histograms (DVHs) for the CTV and PTV of the clinical case under the three HU override scenarios described in Section 2.3. Compared to the plan in which the delineated fiducial marker was overridden with the proposed weight HU method, the V_100%_ of the PTV was 19% lower in the simple overridden plan in which the delineated fiducial structure was entirely overridden with gold material and 1.9% lower in the default non‐overridden HU plan, while the V_100%_ of the CTV was 21.6% and 0.8% lower in the simple overridden HU plan and default non‐overridden HU plan, respectively. No significant difference in the dose coverage of nodal PTV (prescribed to 52.5 Gy) and the maximum dose was observed across the three plans. As an example, Figure 3a,b,c illustrates the corresponding isodose distributions on a few CT slices for one of the three fiducial markers in the TPS. Due to the presence of the gold fiducial markers, distortion of the isodose lines near the markers was evident in the non‐override plan and was more pronounced in the simple overridden plan, indicating different approaches in addressing the artifacts of the gold fiducial markers did contribute to variation in dose distributions and target coverage.

**FIGURE 3a:**
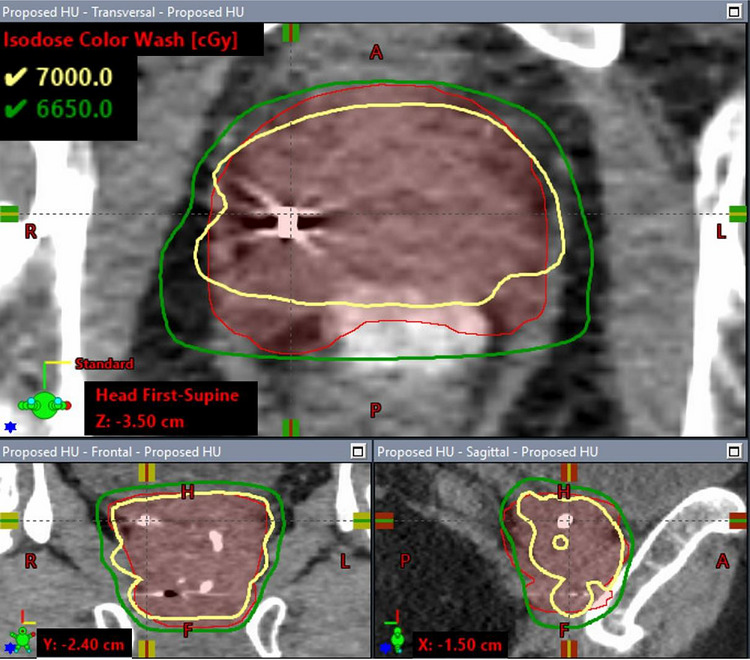
Dose distributions calculated with the proposed HU overridden.

**FIGURE 3b:**
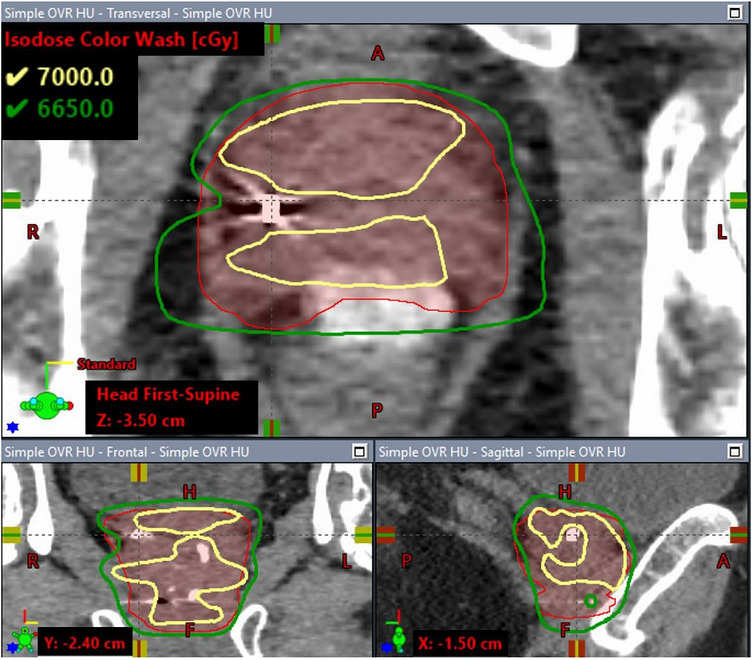
Dose distributions calculated with the simple overridden HU.

**FIGURE 3c:**
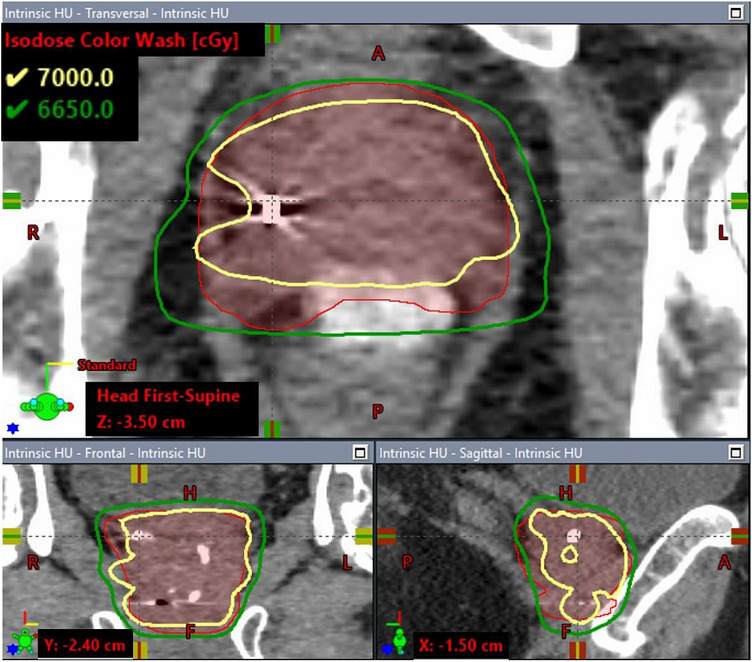
Dose distributions calculated with the default intrinsic HU.

### 3.2 Validation of the robustness of the proposed stopping power calculation

Figure 3d shows the isodose distributions for the reference plan (fiducial markers modeled with manufacturer‐specified dimensions and assigned with 10 000 HU). The respective dose distributions on same CT slices shown in Figures 3a–c are presented in Figure 3d for a direct visual comparison. No significant difference was observed between the two plans in either the isodose distributions or the target coverage. The comparison shows that dose distributions calculated with our proposed method agreed with the near actual situation in which the actual dimensions and orientations of the fiducial markers were modeled in TPS.

**FIGURE 3d:**
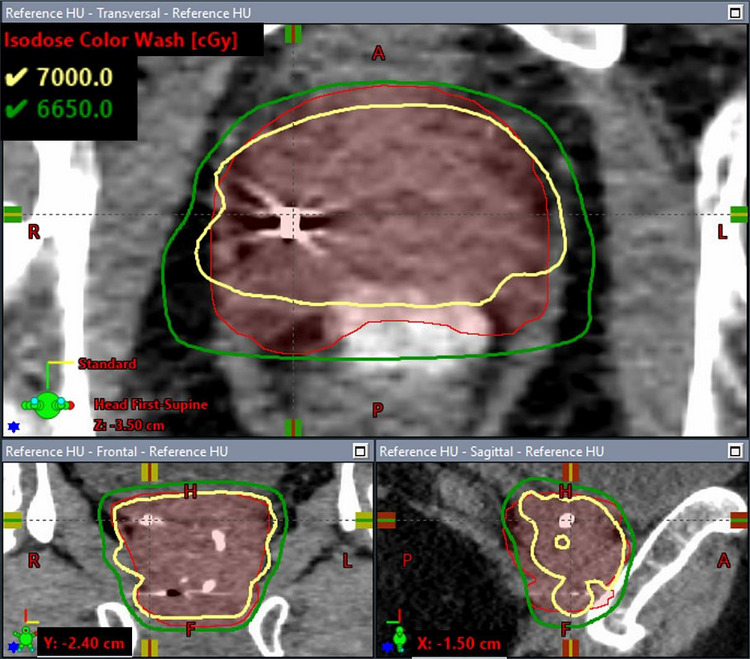
Dose distributions calculated with the reference plan.

Table 3 summarizes the RSP values, their corresponding HU values, and the nominal beam ranges (defined by 90% dose at distal falloff), calculated using the delineated fiducial contours with the enclosed prostate tissue modeled as water, striated muscle, or adipose tissue, respectively. The table also presents the results with those obtained from the delineated fiducial contours expanded by a 1 mm margin. All the RSP values were computed for a 200 MeV proton beam. It was found that the variations in the computed nominal beam range were negligible across the three different tissues. As expected, the fiducial structure with 1‐mm margin led to considerably different RSP and HU values; however, no statistical difference was observed in the target coverage comparing to the plan calculated using the proposed HU. These results indicate that the proposed HU overridden approach in this study may be insensitive, in terms of impacts to proton beam range and target coverage, to the delineation variations introduced by user‐dependent contouring variability and window‐level settings.

**TABLE 3:** Relative stopping power, HU, and nominal beam range for fiducial contours across different surrounding tissues and for contours with a 1 mm margin, calculated with a 200 MeV proton beam.

	RSP	HU	Range field 1 (cm)	Range field 2 (cm)
Fiducial contour with water	1.608	1100	25.02	25.48
Fiducial contour with striated muscle	1.647	1175	25.02	25.50
Fiducial contour with adipose tissue	1.535	960	25.02	25.48
Fiducial contour with 1 mm margin and water	1.163	265	25.02	25.48

## 4 DISCUSSION

The findings of this study demonstrated that selecting an appropriate HU override for gold fiducial markers is important for accurate dose distribution calculation in proton radiation therapy, in the cases where the fiducial markers are present. Because proton range is sensitive to stopping power variations,^11^ inaccuracies in the assigned HU value for high‐*Z* implants can produce measurable distortions from reality in dose distributions, as evidenced by the substantial CTV and PTV under‐dosage observed when a simple HU override (fully gold) was applied to the delineated fiducial structures as they were most likely larger than the actual markers. The default approach (non‐overridden) also introduced dose discrepancies, though to a lesser degree. These results demonstrated that a consistent and reproducible method is needed to ensure accurate representation of the delineated high‐*Z*, mixed‐material structures. This work presents such a method by explicitly deriving the fiducial‐and‐tissue mass fractions and calculating the corresponding RSP based on the actual physical information of the markers and the structure information directly available from TPS. We presented a transparent and clinically practical HU override strategy.

It is observed that the differences in the PTV coverage between the proposed HU overridden and non‐overridden plans were small, whereas the differences between the proposed HU overridden and the simple HU overridden plans were substantial. This is possibly because the mean intrinsic HU value of the materials enclosed inside the delineated fiducial structures without any HU override (2105 HU) was more comparable to the HU (1100 HU) derived with the proposed method than the simple overridden HU value (10000 HU) to the structures. This finding may indicate that the Philips Big Bore RT scanner used in this study is capable of handling high density materials, such as gold, without severe CT number saturation, allowing the raw HU values to be directly used for the stopping power determination for small implants. However, this observation may be scanner specific, as CT number saturation is commonly observed for high density materials when the scanner has a limited dynamic range. In such cases, the intrinsic mean HU value of the material may deviate substantially from its true HU value, which can lead to inaccuracies in dose calculation. Furthermore, when very small implants are present, the partial volume effect can be significant for contouring, leading to unreliable intrinsic HU values. The dose distribution should be carefully evaluated in addition to the DVH evaluation as the distortion of the isodose lines can be present near the fiducial markers as well as other tissue artifacts on the CT images caused by the presence of the fiducial markers. Caution must be exercised when applying manual HU or density overrides, as the partial‐volume or volume‐averaging effect can substantially distort the effective material composition within a contoured region, leading to potentially large deviations in calculated dose.

In addition, the compensator design is directly influenced by the assigned HU along the beam path in double‐scattering proton therapy. When high‐*Z* fiducial markers are inaccurately represented, for example, by assigning the entire contoured volume as pure gold, the resulting stopping power can be significantly overestimated within a small region. This may introduce sharp local gradients in the compensator design near the fiducial location, leading to unnecessary complexity in compensator fabrication and unintended overshoot of distal falloff. In contrast, the proposed HU override approach provides a more realistic representation of the mixed material composition within the delineated fiducial structure, resulting in smoother compensator design with less steep gradients.

A proton energy of 200 MeV was selected as the representative proton energy for the stopping power calculations because, within the clinically relevant energy window for prostate treatments, the relative mass stopping power of gold showed minimal variation.^16^ As presented in Table 2a and b, using 200 MeV produced nearly identical RSPs and HU overrides to those that would be obtained using other viable therapeutic energies. Therefore, the resulting dose distributions are not strongly dependent on the representative proton energy assumed for stopping power calculations, and 200 MeV is an acceptable choice for this purpose.

The robustness of the proposed method for RSP calculation was evaluated by comparing the dose distribution computed using the proposed HU assignment with that obtained using a reality‐based reference representation of the fiducial markers, in which the actual geometry, orientation, location and physical density were exactly modeled and preserved in TPS. Although physical measurements with the actual seed could provide additional validation of local dose perturbations around the metal markers, phantom measurements around millimeter scale fiducial markers in double scattering proton beams are technically challenging, especially due to the positioning uncertainties relative to the steep dose gradients near the metal tissue interface. Minimal differences between the two plans were observed, indicating that our proposed method for fiducial marker HU calculation can assimilate the RSP behavior of the fiducial markers with excellent accuracy and precision for the purpose of dose calculation.

The window‐level setting used during the delineation can affect both the apparent geometry of a small high‐*Z* implant and the amount of adjacent tissue included in the delineated structures. Since our method explicitly accounts for the mass fraction of gold versus that of the surrounding tissue enclosed inside the delineated fiducial structure, therefore any window/level setting that expands or shrinks the contoured volume can alter the mass fraction and hence the mass‐weighted stopping power. The robustness test results presented in Table 3 (adding an isotropic 1‐mm margin to the fiducial structure contours) showed minimal changes in dose distribution and nominal beam range, which indicates that the mass‐weighting approach can mitigate some contouring‐related variability caused by the window‐level settings as well as different users. Although isotropic contour expansion was used in this study, true inter‐user variability may result in non‐uniform contour differences. Since the proposed method is primarily dependent on the contour volume used for RSP/HU assignment, moderate variations in contour shape are expected to have limited dosimetric impact.

We explicitly evaluated three surrogates for the tissue component (adipose, water, and striated muscle). Per PSTAR,^16^ the total mass stopping powers differ only modestly among the three: 5.035 (adipose), 4.903 (water), and 4.861 MeV·cm^2^/g (muscle) for the evaluated proton energy. These small differences produced small changes in the final RSP for the contoured structure and correspondingly negligible differences in nominal beam range in our clinical plan (Table 3). Therefore, using water as the surrogate for tissue within the fiducial contours is acceptable for these calculations.

A recent paper from Vilches‐Freixas et al.^14^ examined strategies for handling implants of a broad range of sizes and materials and compared HU‐override scenarios for small titanium and tantalum clips. That study compared dose distributions for clips (a) overridden to be entirely metal (similar to our “incorrect HU” scenario), (b) contoured too large and overridden to be entirely metal, and (c) not overridden. They acknowledged that blooming artifacts inflate the apparent size of the clips, an effect greatly dependent on the window‐level settings. Since their HU overrides assumed that the entire contour volume consists of the clip material, overestimating the contour size has a considerable impact on dose distribution. In contrast, our work presents a method that accounts for the mixture of materials (metal and tissue) within the delineated implant structures. Therefore, our method mitigates the effect of overestimating implant size. They conclude that proton beams passing through such implants should be avoided.^14^ However, that is not possible in all cases; thus, it is important to have a method readily available to account for their presence without significant loss of accuracy.

This study has some limitations that should be considered. The mass‐weighted RSP calculation proposed in this work provides a physically consistent representation of the effective material composition within the delineated fiducial structure and therefore can be directly incorporated into pencil‐beam scanning (PBS) treatment planning workflows. However, outcomes for PBS scanning could differ from the double scattering system as the PBS system is more sensitive to small‐scale heterogeneities. In addition, potential small dose perturbations at the metal‐tissue interface may not be fully considered by our method using PCS algorithm. Therefore, performing a full Monte Carlo dose calculations will be valuable to capture more intricate dose distribution information. Also, there can be some CT scanner‐dependent effects on the artifacts and HU numbers observed in the vicinity of these implants. Future work can address these limitations by conducting a similar comparison on PBS systems, evaluating this method on scans acquired from different models of CT scanners, performing full Monte Carlo dose calculations or phantom measurements to capture more intricate dose distribution information, and validate the proposed method on a variety of implant types and materials.

## 5 CONCLUSION

We present a method using delineated fiducial structure as the representation of metallic fiducial marker and the mass fractions of fiducial and tissue materials enclosed inside the structure to account for the mismatch between the exact physical dimension of fiducial marker and the corresponding structure to calculate the proton RSP in proton therapy. Using the corresponding HU override in clinical TPS dose calculations resulted in comparable dose coverage of CTV/PTV as compared to idealistic situation where the actual dimensions and orientations of the fiducial markers were modeled in TPS. The method was shown to be robust to contouring variability. The proposed framework can be adapted to other small metallic implants by incorporating their physical density, geometry, and material stopping power properties.

## AUTHOR CONTRIBUTIONS

Xiao Wang, Ning J Yue and Xinxin Zhang conceived of the presented idea. Xiao Wang, Matthew Andriotty and Xinxin Zhang led the computation, analysis and writing of the manuscript. All authors reviewed and approved the final manuscript.

## CONFLICT OF INTEREST STATEMENT

The authors declare no conflicts of interest.
